# LY294002 and LiCl Mitigate Neonatal ExPEC Meningitis Through Akt/GSK3β Signaling Modulation

**DOI:** 10.1155/mi/5086440

**Published:** 2026-03-23

**Authors:** Peicen Zou, Ruiqi Xiao, Sihan Sheng, Peipei Zhang, Pan Huang, Yue Du, Ying Chen, Yajuan Wang

**Affiliations:** ^1^ Capital Institute of Pediatrics, Chinese Academy of Medical Sciences and Peking Union Medical College, Beijing, 100020, China, cacms.ac.cn; ^2^ Department of Neonatology, Capital Center for Children’s Health, Capital Medical University, Capital Institute of Pediatrics, Beijing, 100020, China, shouer.com.cn

**Keywords:** Akt/GSK3β pathway, blood–brain barrier, ExPEC, neonatal purulent meningitis, neuroinflammation

## Abstract

**Background:**

Neonatal purulent meningitis (NPM) is a life‐threatening condition associated with high mortality rates and a substantial risk of severe long‐term neurological sequelae among survivors. Extraintestinal pathogenic *Escherichia coli* (ExPEC) is the predominant causative agent of NPM and exhibits multidrug resistance. Targeting host signaling pathways is a promising therapeutic approach. In this study, we aimed to evaluate the therapeutic potential of LY294002 (a PI3K/Akt inhibitor) and lithium chloride (LiCl) (a GSK3β inhibitor) in a neonatal mouse model of ExPEC meningitis.

**Methods:**

Neonatal mice were infected with clinical ExPEC isolates to induce meningitis. LY294002 or LiCl was administered as an intervention. Brain bacterial load was quantified via culture, while inflammation was assessed using RT‐qPCR to evaluate the expression of inflammatory cytokines; hematoxylin–eosin (HE) staining was also performed. The expression and localization of tight junction (TJ) proteins were analyzed using immunohistochemistry, and western blotting was used to quantify TJ proteins and key signaling molecules, including Akt, phosphorylated Akt (p‐Akt Ser473), GSK3β, and phosphorylated GSK3β (p‐GSK3β Ser9).

**Results:**

ExPEC colonization in the brain tissue was confirmed via bacterial culture. Early intervention with LiCl significantly reduced bacterial load. HE staining revealed meningeal thickening in infected mice, which was alleviated by both LY294002 and LiCl treatments. Western blotting and immunohistochemistry consistently demonstrated a marked reduction in the expression of TJ proteins following ExPEC infection, and their levels were substantially restored by both the interventions. These protective effects were associated with the modulation of the Akt/GSK3β signaling pathway.

**Conclusion:**

LY294002 and LiCl reduced neuroinflammation and preserved the blood–brain barrier (BBB) integrity in the neonatal ExPEC meningitis model, likely via modulation of the Akt/GSK3β pathway. These results underscore the potential of this pathway as a therapeutic target and provide a basis for further preclinical investigation.

## 1. Introduction

Neonatal purulent meningitis (NPM), a serious infectious disease of the central nervous system (CNS) during the neonatal period, represents a major cause of neonatal mortality and long‐term neurological sequelae [1]. The incidence of NPM varies substantially across regions. In low‐income countries, it affects ~0.8%–6.1% of live births, with mortality rates as high as 40%–58% [2]. Approximately 27%–50% of survivors develop long‐term severe neurological sequelae, including intellectual disability, motor dysfunction, epilepsy, hearing loss, and sensory deficits, posing substantial challenges to affected children and their families [3, 4]. Early‐onset NPM occurs primarily via vertical transmission. The mother‐to‐child transmission rate of extraintestinal pathogenic *Escherichia coli* (ExPEC) is ~17%. Perinatal risk factors, including premature rupture of membranes, prolonged labor, and dystocia, considerably increase the risk of neonatal infection [5, 6]. ExPEC has emerged as the major pathogen in NPM and exhibits multidrug resistance [7, 8].

The blood–brain barrier (BBB) is a critical structural and functional interface in the brain that maintains homeostasis, regulates nutrient transport, and restricts the entry of macromolecules and pathogens into the CNS. The maintenance of its integrity is critical to protect the body from neurological diseases [9, 10]. Tight junction (TJ) proteins primarily include claudins, occludins, the zonula occludens (ZO) family members, and junctional adhesion molecules. These proteins, together with brain microvascular endothelial cells, basement membrane, astrocytes, and pericytes, form the BBB and play an important role in maintaining the integrity of the barrier [11, 12]. Upon colonizing the neonatal intestinal mucosal barrier, ExPEC crosses this mucosal barrier and enters the bloodstream, where it proliferates and induces a high level of bacteremia. This process disrupts the expression of TJ proteins, compromises the BBB integrity, and increases vascular permeability, ultimately leading to CNS infection in neonates [13, 14].

LY294002 is a broad‐spectrum PI3K inhibitor that blocks Akt activation and suppresses its downstream antiapoptotic effects. Infections, injuries, and other stimuli can trigger inflammatory responses in the human body; however, the regulation of inflammation is an extremely complex process. Ma et al. [15] found that LY294002 can reduce the inflammatory response of the bronchi and blood vessels in mice with allergic asthma by inhibiting the activation of the PI3K/Akt signaling pathway. Lithium chloride (LiCl), a mood stabilizer clinically used to treat bipolar disorder, also exhibits neuroprotective properties. It inhibits GSK3β phosphorylation by competing with the magnesium ion of GSK3β, thereby activating the Wnt/β‐catenin signaling pathway and participating in the development of the early nervous system [16]. In vitro and in vivo studies have shown that LiCl reduces LPS‐induced retinal inflammation, suppresses pro‐inflammatory cytokine production, promotes neuronal survival, and inhibits apoptosis [17].

Although LY294002 and LiCl have each been studied as modulators of Akt and GSK3β signaling, respectively, in various inflammatory models, their effects in neonatal ExPEC meningitis have not yet been explored [18, 19]. Our previous in vitro study findings suggest that lipopolysaccharide can disrupt TJ proteins via Akt activation in brain microvascular endothelial cells, supporting a potential role of the PI3K/Akt pathway in BBB injury [20]. Based on this observation, we hypothesized that by modulating the Akt/GSK3β signaling pathway, the BBB integrity can be preserved and neuroinflammation can be mitigated during ExPEC meningitis. Therefore, in this study, we aimed to evaluate the therapeutic effects of LY294002 and LiCl in a neonatal mouse model of ExPEC meningitis and explore the underlying pathogenic mechanisms.

## 2. Materials and Methods

This study was conducted at the Capital Institute of Pediatrics (Beijing, China) between April 2023 and May 2024. All animal procedures were approved by the Ethics Committee for Experimental Animals of the Capital Institute of Pediatrics (Approval Number DWLL2023012) and were conducted in accordance with the ARRIVE 2.0 guidelines.

### 2.1. Bacterial Culture

The *E. coli* strain used in this study was isolated from the cerebrospinal fluid of an infant with meningitis [8]. Whole‐genome sequencing data have been deposited in the Genome Warehouse of the National Genomics Data Center under Accession Number GWHGGFB00000000.1; they are publicly accessible at https://ngdc.cncb.ac.cn/gwh [21, 22]. Virulence gene profiling was performed using ABRicate v1.0.1 with the VFDB database (release: Jan 14, 2025) with default parameters (≥80% identity and ≥80% coverage).

Bacteria were cultured overnight on Luria–Bertani (LB) agar plates and then inoculated into LB broth and incubated at 37°C with shaking at 200 rpm until the culture reached the logarithmic growth phase. Bacterial concentration was determined using both optical density at 600 nm (OD_600_), measured via spectrophotometry, and serial dilution plating for colony counting.

### 2.2. Model Optimization for *E. coli* Meningitis

Neonatal C57BL/6 mice (5 days old; sex not distinguished owing to the technical difficulty of accurate sex determination at this age) were purchased from Beijing Weitong Lihua Laboratory Animal Technology (Beijing, China) and maintained under standard specific pathogen‐free conditions. To determine the optimal infectious dose and duration for model establishment, neonatal mice (*n* = 3 per group per condition) were injected intraperitoneally with *E. coli* at various concentrations (10^1^ to 10^7^ colony forming units [CFU]/mL) and evaluated at different postinfection intervals (2–10 h). The brain tissue cultures were conducted to confirm successful colonization. Based on pilot study results, 10^6^ CFU/mL with a 6‐h infection duration was selected for subsequent experiments.

### 2.3. *Escherichia coli* Meningitis Mouse Model

Neonatal C57BL/6 mice (*n* = 3 mice per group) were randomly assigned to four groups based on a simple randomization method: *E. coli*, *E. coli* + LY294002, *E. coli* + LiCl, and control. For the infection model, 10^6^ CFU/mL of *E. coli* was administered intraperitoneally, and blood and brain tissues were collected for culture after 6 h. Mice in the intervention groups received intraperitoneal injections of LY294002 (40 μM, 10 μL) or LiCl (3 mmol/kg) 1 h prior to infection [23, 24]. No overt signs of toxicity, mortality, or abnormal behavior were observed in the neonatal mice throughout the experimental period. 6 h after the bacterial injection into the abdominal cavity, all animals were anesthetized with isoflurane. To exclude the influence of circulating bacteria, the neonatal mice were perfused with sterile 0.9% saline via the ventricle prior to tissue collection.

### 2.4. Brain Histopathology

At 6 h postinfection, the mice were euthanized, followed by collection of the brain and fixation in 4% paraformaldehyde for at least 24 h. Paraffin‐embedded brain tissues were sectioned at a thickness of 4 μm. The sections were deparaffinized, rehydrated, and stained with hematoxylin and eosin (H&E). Histological changes were observed and photographed using a light microscope by investigators blinded to group allocation.

### 2.5. mRNA Expression of IL‐1β, IL‐6, and TNF‐α in the Brain Tissue

Total RNA was extracted from the brain tissue using the Mini BEST Universal RNA Extraction Kit (TaKaRa), and RNA purity and concentration were assessed using a NanoDrop 2000 spectrophotometer (Thermo Fisher, USA). cDNA synthesis was performed using a 5× All‐in‐One RT MasterMix kit (ABM, Canada). RT‐qPCR was carried out using the BlasTaq 2× qPCR MasterMix (ABM) on a QuantStudio Real‐Time PCR System (Applied Biosystems, USA). The amplification protocol was as follows: hold at 50°C for 2 min and 95°C for 3 min; 40 PCR cycles at 95°C for 15 s and 60°C for 1 min; and melting curve analysis at 95°C for 15 s, 60°C for 1 min, and 95°C for 15 s. qPCR data analysis was conducted by investigators blinded to sample identity. The primer sequences are listed in Table 1.

**Table 1:** Primer sequences used for RT‐qPCR.

Gene	Primer type	Sequences
*IL-6*	Forward	GAGAGGAGACTTCACAGAGGATACC
	Reverse	TCATTTCCACGATTTCCCAGAGAAC
*IL-1*β	Forward	TCGCAGCAGCACATCAACAAG
	Reverse	CCAGCAGGTTATCATCATCATCCC
*TNF-α*	Forward	ACGCTCTTCTGTCTACTGAACTTCG
	Reverse	TGGTTTGTGAGTGTGAGGGTCTG
*GAPDH*	Forward	ACTCCACTCACGGCAAATTCAAC
	Reverse	ACACCAGTAGACTCCACGACATAC

### 2.6. Western Blotting

To assess the expression and phosphorylation levels of key signaling and TJ proteins in the brain tissue, total protein was extracted from the brain tissue on ice using RIPA lysis buffer supplemented with phosphatase/protease inhibitors, including phenylmethylsulfonyl fluoride (Solepol, China). Protein concentration was measured using a BCA protein assay kit (Beyotime, China). Samples were denatured at 95°C for 10 min and separated using SDS–PAGE, followed by transfer onto polyvinylidene membranes. The membranes were blocked and incubated with primary antibodies overnight at 4°C, and then with secondary antibodies. The antibodies used were ZO‐1 (1:500, Invitrogen), occludin (1:1000, CST), claudin‐5 (1:1000, Invitrogen), Akt (1:1000, CST), p‐Akt (Ser473) (1:1000, CST), GSK3β (1:1000, CST), p‐GSK3β (Ser9) (1:1000, CST), and β‐actin (1:1000, Proteintech). Signal detection was performed using enhanced chemiluminescence, and band intensity was quantified using the ImageJ software (NIH, USA). Protein levels were normalized to the level of β‐actin. Protein quantification was performed by investigators blinded to group allocation.

### 2.7. Immunohistochemistry

To visualize the localization and expression of TJ proteins in situ, paraffin‐embedded sections were deparaffinized, rehydrated, and subjected to antigen retrieval in 0.01 M citrate buffer (pH 6.0) under microwave heating. Endogenous peroxidase activity was quenched, and tissue permeabilization was performed using 0.3% Triton X‐100. The sections were blocked with 5% goat serum for 1 h at room temperature, followed by overnight incubation at 4°C with the following primary antibodies: ZO‐1 (1:500, Servicebio), occludin (1:700, Servicebio), and claudin‐5 (1:700, Servicebio). After washing, the sections were incubated with horseradish peroxidase‐conjugated secondary antibodies for 30 min. Color development was performed using 3,3^′^‐diaminodbenzidine substrate, and the nuclei were counterstained with hematoxylin. The sections were dehydrated, cleared, and mounted with neutral resin for microscopic observation. Image evaluation was performed by investigators blinded to the grouping.

### 2.8. Statistical Analysis

All experiments were performed in triplicate. Data are presented as mean ± standard deviation (SD). Statistical analyses and preparation of graphs were performed using R 4.3.1 and GraphPad Prism 10.0. One‐way ANOVA followed by Tukey’s multiple comparisons test was used to evaluate group differences. Results with a *p*‐value of <0.05 were considered statistically significant.

## 3. Results

### 3.1. Animals and Bacterial Characteristics

A total of 42 neonatal C57BL/6 mice (5 days old, average weight 2.3–3.0 g) were used across all experiments. No obvious differences in baseline body weight or condition were observed among the groups. The clinical ExPEC isolate used for model induction was characterized via whole‐genome sequencing and virulence profiling, which revealed the presence of several meningitis‐associated virulence genes, including *ompA*, but *ibeA* and *cnf1* were not detected. A full list of the identified virulence factors is provided in Table S1.

### 3.2. Optimizing Dose and Duration for Establishing the *E. coli* Meningitis Model

To establish a reliable model of neonatal meningitis, we evaluated the effects of different bacterial doses and infection durations. Neonatal mice were injected with *E. coli* at concentrations ranging from 10^1^to10^7^ CFU/mL and euthanized 8 h postinfection. Bacterial cultures from brain homogenates confirmed that *E. coli* could be consistently recovered when mice were exposed to *E. coli* at concentrations of 10^6^ CFU/mL or above (Figure 1).

Figure 1Dose and time optimization for neonatal *Escherichia coli* meningitis model. (A) Brain culture results following infection with *E. coli* at different concentrations (0–10^7^ CFU/mL) at 8 h postinfection. (B) Time‐course of brain colonization at different time points following infection with 10^7^ CFU/mL *E. coli* (0–10 h). Bacterial counts are presented as log_10_ CFU/g of the brain tissue. *Escherichia coli* was consistently recovered from the brain tissue when mice were infected with ≥10^5^ CFU/mL for 8 h or 10^7^ CFU/mL for 4 h. CFU, colony‐forming unit.

**Figure:**
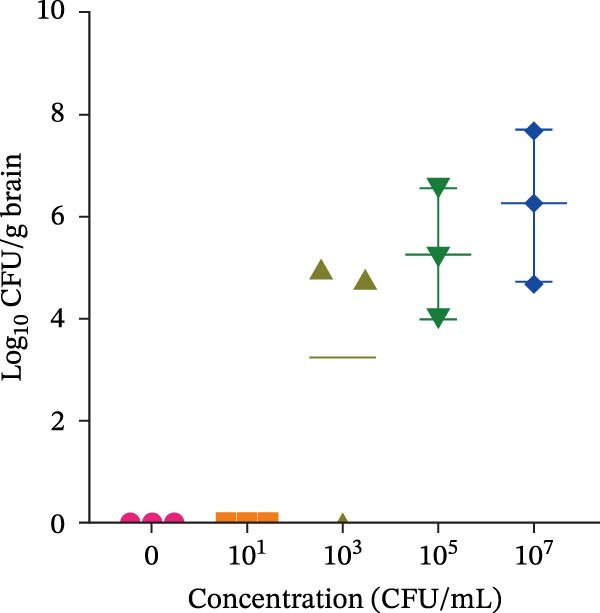
(A)

**Figure:**
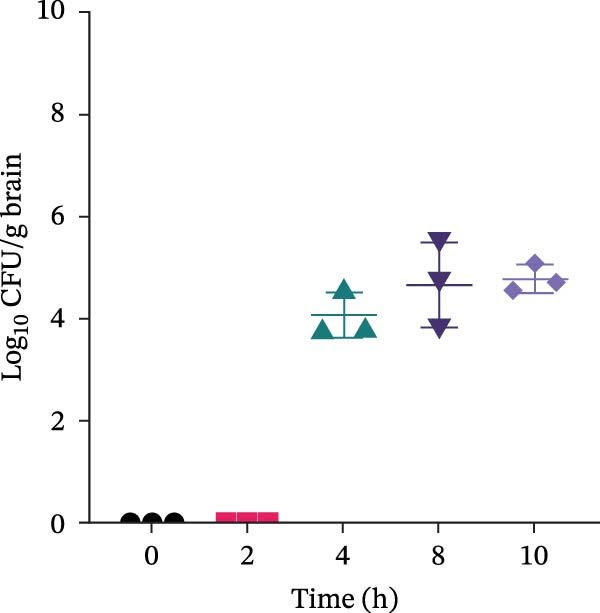
(B)

In a separate set of experiments, mice infected with a high dose (10^7^ CFU/mL) were observed over a 10‐h period. At 4 h, colonization began to appear, whereas at 8 h, severe clinical symptoms emerged. By 10 h, mortality occurred. Brain cultures confirmed increasing bacterial burden over time (Figure 1).

Based on the above‐mentioned results, an inoculum of 10^6^ CFU/mL with a 6‐h infection duration was selected for subsequent experiments, as it consistently yielded brain colonization with minimal early‐stage mortality.

### 3.3. LiCl Reduced *E. coli* Colonization in the Brain Tissue of Neonatal Mice With Meningitis

**Figure 2:**
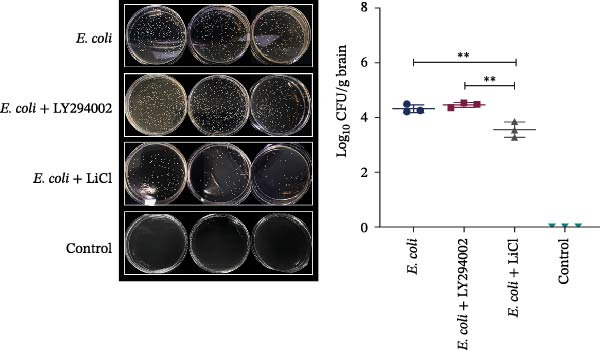
LiCl reduced *Escherichia coli* colonization in neonatal mouse brain tissue. Neonatal mice were infected with *E. coli* and treated with LY294002 or LiCl as indicated. The bacterial load was normalized per gram of the brain tissue and log_10_‐transformed for analysis. LiCl significantly reduced the bacterial load in the brain tissue (*p* = 0.0066), whereas LY294002 had no effect (*p* = 0.6662).  ^∗∗^ *p* < 0.01 vs. *E. coli* group.

In the brain tissue cultures, negative results for bacterial growth were obtained only for the control group; all other groups showed positive bacterial growth. LY294002 treatment did not result in a significant reduction in bacterial counts. Notably, the *E. coli* + LiCl group exhibited a significantly reduced bacterial colony count than the *E. coli* group, indicating that LiCl treatment effectively reduced bacterial load in the brain tissue (Figure 2).

### 3.4. LY294002 and LiCl Attenuated the Expression of Inflammatory Cytokines and Histopathological Changes

The RT‐qPCR analysis showed that the mRNA levels of IL‐6, IL‐1β, and TNF‐α considerably increased following *E. coli* infection. Both LY294002 and LiCl interventions significantly downregulated the expression of these inflammatory cytokines, with LiCl showing a stronger anti‐inflammatory effect than LY294002 (Figure 3A).

Figure 3LY294002 and LiCl attenuated neuroinflammation in neonatal *Escherichia coli* meningitis. (A) mRNA expression of the pro‐inflammatory cytokines IL‐6, IL‐1β, and TNF‐α in neonatal mouse brain tissue was measured using RT‐qPCR. Expression levels were normalized to *Gapdh* level. Both LY294002 and LiCl reduced cytokine induction, with LiCl showing a stronger effect. (B) Representative hematoxylin and eosin‐stained sections of the cerebral cortex and meninges. Meningeal thickening and vascular congestion were evident in the *E. coli* group; however, these pathological features were alleviated following LY294002 or LiCl treatment. (B1–B4) Low magnification (×50), scale bar = 200 μm. (B5–B6) High magnification (×100), scale bar = 100 μm. (B1, B5) *E. coli*; (B2, B6) *E. coli* + LY294002; (B3, B7) *E. coli* + LiCl; (B4, B8) control. Black arrows indicate the meninges.  ^∗∗^ *p* < 0.01,  ^∗∗∗∗^ *p*  < 0.000 vs. *E. coli* group.

**Figure:**
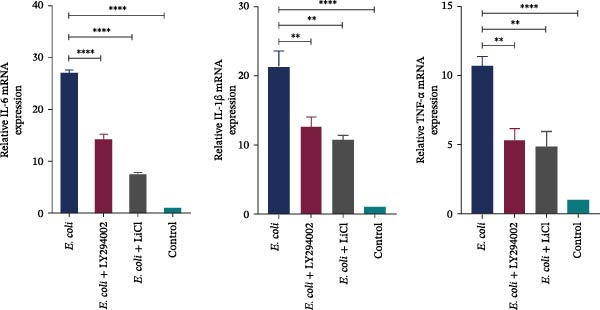
(A)

**Figure:**
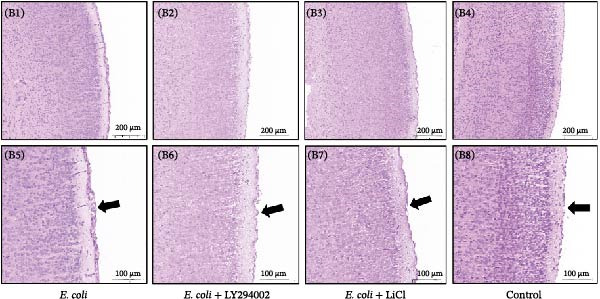
(B)

H&E staining revealed pronounced thickening and congestion of the meninges in the *E. coli* group. In contrast, mice treated with LY294002 or LiCl displayed only mild meningeal thickening, which may reflect diminished inflammation (Figure 3B).

### 3.5. LY294002 and LiCl Preserved the BBB Integrity in Infected Neonatal Mice

Western blotting demonstrated that levels of the TJ proteins, ZO‐1, occludin, and claudin‐5, significantly decreased after *E. coli* infection compared with the uninfected control group. Immunohistochemistry confirmed decreased expression and altered localization of these proteins, particularly around brain capillaries. Notably, LY294002 and LiCl treatment prevented this decrease and preserved the expression of TJ proteins, indicating a protective effect on the BBB integrity. No significant differences were observed in the expression of any of the TJ markers between the LY294002‐ and LiCl‐treated groups, indicating overall comparable BBB‐protective effects. Collectively, these findings suggest that LY294002 and LiCl partially preserve the BBB TJ integrity following neonatal *E. coli* meningitis (Figure 4A,B).

Figure 4LY294002 and LiCl protected blood–brain barrier integrity via regulation of the Akt/GSK3β signaling pathway. (A) Western blot analysis of the tight junction proteins ZO‐1, occludin, and claudin‐5 in the brain tissue of neonatal mice. Expression of all three proteins significantly decreased following *Escherichia coli* infection, and it was restored upon treatment with LY294002 or LiCl. (B) Representative immunohistochemical staining for ZO‐1, occludin, and claudin‐5 in the brain. In the *E. coli* group, the expression of the tight junction proteins was markedly downregulated around the brain microvasculature, and treatment with LY294002 or LiCl restored their expression and localization. (C) Measurement of the expression of the signaling molecules Akt, p‐Akt, GSK3β, and p‐GSK3β using western blot. Akt phosphorylation increased after *E. coli* infection, and it was inhibited by LY294002. GSK3β phosphorylation was significantly enhanced by LiCl treatment.  ^∗^ *p* < 0.05,  ^∗∗^ *p* < 0.01.

**Figure:**
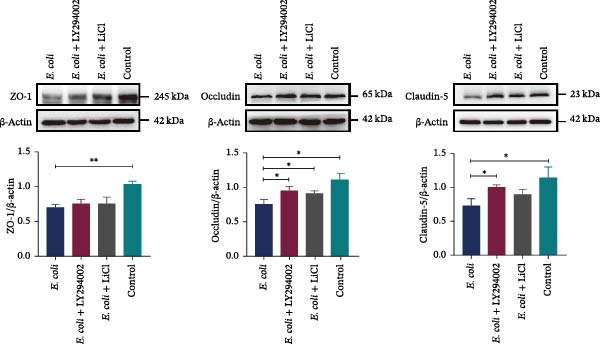
(A)

**Figure:**
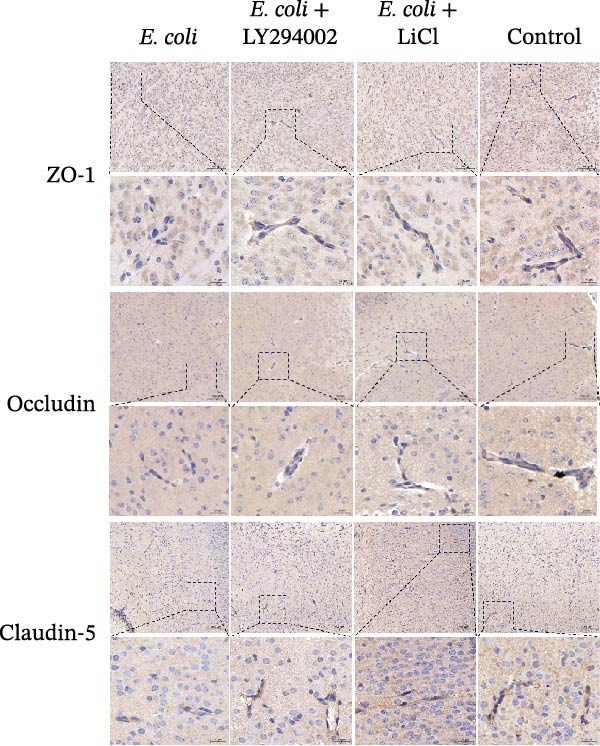
(B)

**Figure:**
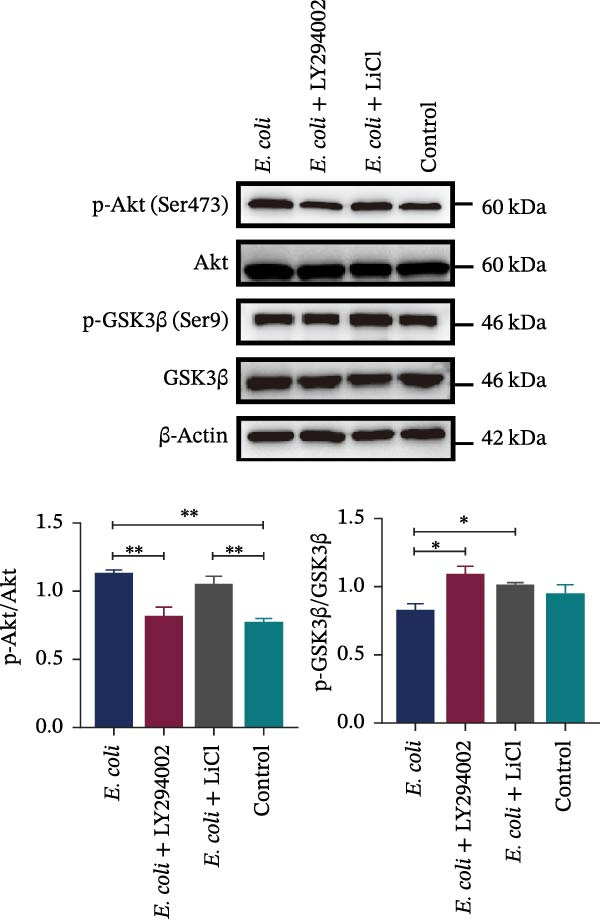
(C)

### 3.6. LY294002 and LiCl Exerted Effects Through the Akt/GSK3β Signaling Pathway

**Figure 5:**
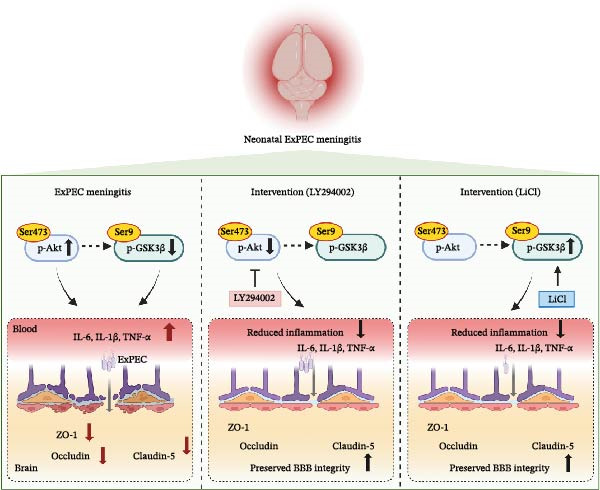
Proposed model of Akt/GSK3β‐targeted interventions for neonatal ExPEC meningitis. ExPEC infection elevated Akt phosphorylation at Ser473 (p‐Akt Ser473) and showed a mild decreasing trend in inhibitory phosphorylation of GSK3β at Ser9 (p‐GSK3β Ser9). These changes were accompanied by increased pro‐inflammatory cytokines (IL‐6, IL‐1β, and TNF‐α) and decreased levels of tight‐junction (TJ) proteins (ZO‐1, occludin, and claudin‐5), consistent with blood–brain barrier (BBB) disruption. Treatment with LY294002 (a PI3K/Akt inhibitor) suppressed Akt phosphorylation and attenuated cytokine induction, whereas LiCl increased inhibitory phosphorylation of GSK3β at Ser9 (p‐GSK3β Ser9). Both interventions partially preserved TJ proteins and BBB integrity. Collectively, these findings support that LY294002 and LiCl confer BBB protection and anti‐inflammatory effects through modulation of the Akt/GSK3β pathway at its upstream (Akt) and downstream (GSK3β) nodes, respectively. Dashed arrows indicate that changes in Akt and GSK3β phosphorylation were not fully coupled under infection conditions.

The western blot analysis showed that the phosphorylation of Akt at Ser473 (p‐Akt Ser473) significantly increased in the brain tissue following *E. coli* infection. Treatment with LY294002 effectively reduced p‐Akt (Ser473) levels to near‐control values. In contrast, the phosphorylation of GSK3β at Ser9 (p‐GSK3β Ser9)—a modification known to inhibit the GSK3β activity—did not significantly change after infection alone but was markedly elevated following LiCl treatment (Figure 4C). These results suggest that LY294002 and LiCl exert their effects by modulating the Akt/GSK3β pathway. A proposed schematic diagram summarizing the mechanism is provided in Figure 5.

## 4. Discussion

Bacterial meningitis caused by ExPEC remains a serious clinical challenge, particularly in the context of increasing antimicrobial resistance. The aim of this study was to establish a neonatal ExPEC meningitis model and to determine whether targeting the Akt/GSK3β pathway alleviates neuroinflammation and the BBB injury. The five major findings are as follows. First, we identified dose–time conditions that reliably induced brain colonization while limiting early mortality. Second, LiCl significantly reduced the brain bacterial burden, whereas LY294002 did not have a significant effect on bacterial load. Third, both LY294002 and LiCl attenuated infection‐induced cytokine upregulation and pathological changes in the meninges. Fourth, both interventions partially preserved the TJ proteins and BBB integrity, with overall comparable BBB‐protective effects between the treatments. Finally, ExPEC infection increased Akt phosphorylation at Ser473, which was inhibited by LY294002. Moreover, LiCl increased the inhibitory phosphorylation of GSK3β at Ser9, supporting the involvement of this signaling axis.

Neonates are a key target population for meningitis prevention and management, and age‐appropriate animal models are essential for mechanistic studies. Previous work has established experimental bacterial meningitis using intracerebroventricular or cisterna magna inoculation, as well as via systemic inoculation routes such as intraperitoneal injection [25, 26]. As our study focused on ExPEC‐associated BBB injury, we adopted a bloodstream‐related infection route to better mimic the clinical progression from neonatal sepsis to meningitis, thereby facilitating investigation of early BBB‐disruptive mechanisms. In addition, preterm infants represent a major at‐risk population, whereas many existing models rely on juvenile or adult animals and may not accurately capture neonatal physiology. Based on the commonly used developmental age‐equivalency estimates, the brain development of postnatal day 1–5 C57BL/6 mice approximates that of humans at 23–32 weeks of gestation; therefore, we used 5‐day‐old neonatal C57BL/6 mice in this study [27, 28].

In our dose‐escalation and time‐course experiments, lower inoculum concentrations caused bacteremia but did not consistently lead to sustained CNS invasion, whereas exposure to higher bacterial concentrations resulted in reproducible detection of CFUs in the brain tissue. A separate high‐dose time‐course experiment further indicated that prolonged exposure increased disease severity and mortality, consistent with prior evidence that meningitis occurrence is closely linked to the bacteremia levels [29]. Based on these data, we selected an inoculum concentration of 10^6^ CFU/mL with a 6‐h infection duration for subsequent experiments to achieve consistent brain colonization while minimizing early mortality. Notably, LiCl significantly reduced brain bacterial burden, whereas LY294002 did not produce a comparable reduction. One possible explanation is that LiCl may indirectly limit bacterial accumulation in the CNS by attenuating inflammation‐associated barrier injury and/or modulating innate immune responses, thereby reducing bacterial entry into, or persistence within, the CNS. In contrast, PI3K/Akt inhibition may preferentially regulate host inflammatory signaling and may not directly affect bacterial growth or clearance within this short observation window. Thus, our study not only optimizes a practical neonatal ExPEC meningitis modeling window but also suggests that targeting GSK3β may influence brain bacterial burden during the acute phase.

ExPEC infection compromises the BBB integrity and triggers inflammatory responses with the release of mediators, such as cytokines and chemokines [30]. In our study, ExPEC infection led to meningeal thickening and significantly increased mRNA levels of IL‐6, TNF‐α, and IL‐1β in neonatal mice, indicating an early inflammatory response in the meninges. A previous study has suggested that excessive production of pro‐inflammatory cytokines is a major contributor to neuronal injury during meningitis [31]. Kim et al. [32] reported that CNS invasion by K1 capsule‐positive ExPEC is not necessarily accompanied by prominent infiltration of inflammatory cells, such as neutrophils and macrophages, which is consistent with our observation of minimal neutrophil presence in the meninges. Notably, early intervention with either LY294002 or LiCl significantly reduced the expression of these cytokines and alleviated meningeal pathological changes on H&E staining, with LiCl showing a more pronounced anti‐inflammatory effect. Collectively, these findings support the notion regarding the Akt/GSK3β axis as a tractable host signaling node that links neuroinflammation to tissue injury in neonatal ExPEC meningitis.

The BBB dysfunction is a central pathogenic event in bacterial meningitis. Pathogens are generally believed to cross the BBB via transcellular transport, paracellular passage, or a “Trojan horse” mechanism [33]. Disruption of TJ complexes is a key mechanism in ExPEC‐induced meningitis pathogenesis [34, 35]. ExPEC induces primary TJ disassembly, enabling paracellular BBB crossing and CNS invasion. Subsequent release of inflammatory cytokines, including IL‐6, IL‐1β, and TNF‐α, further degrades TJ integrity [36]. In our study, the levels of membrane‐localized ZO‐1, occludin, and claudin‐5 markedly decreased after ExPEC infection, supporting an important contribution of paracellular disruption in this model. However, we did not directly assess transcellular transport or Trojan horse mechanisms; therefore, we cannot conclude whether ExPEC uses all three routes under our experimental conditions. Notably, both LY294002 and LiCl partially preserved the expression of TJ proteins and improved their vascular localization patterns. A key novelty of this study is that, in neonatal ExPEC meningitis, targeting upstream Akt activation and downstream GSK3β regulation can confer BBB‐protective effects, supporting the contention that the Akt/GSK3β pathway offers multiple actionable intervention nodes.

Akt is activated via phosphorylation at Ser473, and it can phosphorylate GSK3β at Ser9, leading to its inactivation [37, 38]. In our study, ExPEC infection significantly increased Akt phosphorylation at Ser473 in the neonatal brain tissue, which was reversed by early intraperitoneal administration of LY294002. In contrast, GSK3β phosphorylation at Ser9 showed a mild decreasing trend following infection; however, it markedly increased after LiCl administration. These findings indicate that Akt activation is prominently associated with ExPEC infection, whereas regulation of GSK3β activity through Ser9 phosphorylation appears limited under these conditions. This observation suggests that Akt/GSK3β signaling may be influenced by pathogen‐derived virulence factors, inflammatory signals, or phosphatase activity [39]. Such perturbations could contribute to the BBB dysfunction and inflammatory responses. In our study, independent administration of LY294002 or LiCl alleviated the disruption of the TJ complex and reduced the expression of pro‐inflammatory cytokines. Previous studies have also demonstrated that Akt/GSK3β dysregulation is linked to TJ instability and BBB impairment in intracerebral hemorrhage models [40, 41]. Compared with current BBB‐protection strategies, such as neutralizing antibodies that mainly act on specific inflammatory cytokines, our approach targets upstream and downstream components of the Akt/GSK3β pathway offering broader modulation of both inflammatory signaling and TJ integrity. Moreover, small‐molecule agents, such as LY294002 and LiCl, are relatively accessible and cost‐effective, enabling early intervention. However, further translational studies are needed to evaluate their safety, specificity, and pharmacokinetics in neonatal populations.

Notably, whole‐genome sequencing revealed that the ExPEC strain used in this study harbored the *ompA* gene but lacked other well‐known meningitis‐associated virulence factors, such as *ibeA* and *cnf1*. Previous studies have shown that ompA, an outer membrane protein of *E. coli*, can engage the host receptor gp96 and activate the PI3K/Akt signaling pathway, causing subsequent TJ downregulation of TJ proteins [42, 43]. In vivo studies have shown that *ompA*‐deficient strains exhibit markedly reduced ability to cross the BBB and, consequently, attenuate CNS pathology, suggesting that OmpA may directly contribute to alterations in the BBB permeability [44]. Based on our findings, it is plausible that OmpA‐mediated Akt activation could potentially act synergistically with lipopolysaccharide‐induced signaling to intensify TJ disruption and promote BBB leakage. Although *ompA* was not the focus of this study, its presence in our strain warrants further investigation into its potential contribution to the activation of the Akt pathway and disease progression.

This study has several methodological strengths, including the development of an age‐appropriate neonatal mouse model with a bloodstream‐related infection route and identification of a defined dose–time window that yielded reproducible brain colonization with limited early mortality. One limitation of this study was the lack of sex stratification. The neonatal mouse model used in this study was developed based on established protocols from a previous study, which similarly did not differentiate sex on postnatal day 5 [45]. This approach was adopted owing to the technical challenges of accurately determining sex at this early developmental stage and the minimal physiological differences reported between sexes at this age. In addition, as an acute neonatal mouse model, these findings are preclinical and should not be directly extrapolated to human neonates; further validation in complementary models and human‐relevant systems is warranted.

## 5. Conclusions

This study demonstrated that targeting the Akt/GSK3β axis with LY294002 or LiCl attenuates early neuroinflammation and partially preserves the integrity of the TJ in the BBB using a neonatal ExPEC meningitis model. The observed effects were accompanied by pathway‐specific modulation of Akt and GSK3β phosphorylation. This preclinical work is hypothesis‐generating and warrants further validation and translational evaluation.

## Author Contributions

**Peicen Zou**: conceptualization, data curation, formal analysis, validation, visualization, writing – original draft. **Ruiqi Xiao**: data curation, validation. **Sihan Sheng**: formal analysis, writing – review and editing. **Peipei Zhang**: data curation, supervision. **Pan Huang, Yue Du, and Ying Chen:** supervision. **Yajuan Wang**: conceptualization, funding acquisition, resources, writing – review and editing.

## Funding

This work was supported by the Natural Science Foundation of Beijing Municipality (Grants 7232009 and 7244289), the Cross‐Cooperation Project of Beijing Science and Technology New Star Program (Grant 20240484724), and the High Level Public Health Technical Personnel Construction Project (Grant Subject leaders‐03‐02).

## Ethics Statement

The authors have nothing to report.

## Consent

The authors have nothing to report.

## Conflicts of Interest

The authors declare no conflicts of interest.

## Supporting Information

Additional supporting information can be found online in the Supporting Information section.

## Supporting information


**Supporting Information** Table S1: List of virulence factors identified in the clinical ExPEC isolate using whole‐genome sequencing.

## Data Availability

The data that support the findings of this study are available from the corresponding author upon reasonable request.
