# Supplementary material for: LY294002 and LiCl Mitigate Neonatal ExPEC Meningitis Through Akt/GSK3β Signaling Modulation
Source: Mediators Inflamm. 2026 Mar 23;2026:5086440. doi: 10.1155/mi/5086440 (PMC13140248; doi:10.1155/mi/5086440)
Supplement: Supplementary file 1 — Supporting Information Table S1: List of virulence factors identified in the clinical ExPEC isolate using whole‐genome sequencing. [file MI-2026-5086440-s001.docx]

| Supplementary Table 1 Virulence factors identified in the *E. coli* strain. | | | | |
| --- | --- | --- | --- | --- |
| Sequence | Gene | %coverage | %identity | Product |
| Chromosome_1 | *chuV* | 100 | 99.75 | (chuV) ATP-binding hydrophilic protein ChuV [Chu (VF0227)] [Escherichia coli CFT073] |
| Chromosome_1 | *chuU* | 100 | 99.3 | (chuU) heme permease protein ChuU [Chu (VF0227)] [Escherichia coli CFT073] |
| Chromosome_1 | *chuY* | 100 | 98.4 | (chuY) ChuY [Chu (VF0227)] [Escherichia coli CFT073] |
| Chromosome_1 | *chuX* | 100 | 100 | (chuX) putative heme-binding protein ChuX [Chu (VF0227)] [Escherichia coli CFT073] |
| Chromosome_1 | *chuW* | 100 | 99.25 | (chuW) Putative oxygen independent coproporphyrinogen III oxidase [Chu (VF0227)] [Escherichia coli CFT073] |
| Chromosome_1 | *chuT* | 100 | 99.8 | (chuT) periplasmic heme-binding protein ChuT [Chu (VF0227)] [Escherichia coli CFT073] |
| Chromosome_1 | *chuA* | 100 | 99.54 | (chuA) Outer membrane heme/hemoglobin receptor ChuA [Chu (VF0227)] [Escherichia coli CFT073] |
| Chromosome_1 | *chuS* | 100 | 99.03 | (chuS) heme oxygenase ChuS [Chu (VF0227)] [Escherichia coli CFT073] |
| Chromosome_1 | *gspC* | 100 | 94.58 | (gspC) general secretion pathway protein C [T2SS (VF0333)] [Shigella dysenteriae Sd197] |
| Chromosome_1 | *gspD* | 100 | 96.06 | (gspD) general secretion pathway protein D [T2SS (VF0333)] [Shigella dysenteriae Sd197] |
| Chromosome_1 | *gspE* | 100 | 94.85 | (gspE) general secretion pathway protein E [T2SS (VF0333)] [Shigella dysenteriae Sd197] |
| Chromosome_1 | *gspF* | 100 | 94.17 | (gspF) general secretion pathway protein F [T2SS (VF0333)] [Shigella dysenteriae Sd197] |
| Chromosome_1 | *gspG* | 100 | 95.83 | (gspG) general secretion pathway protein G [T2SS (VF0333)] [Shigella dysenteriae Sd197] |
| Chromosome_1 | *gspH* | 100 | 96.61 | (gspH) general secretion pathway protein H [T2SS (VF0333)] [Shigella dysenteriae Sd197] |
| Chromosome_1 | *gspI* | 100 | 94.62 | (gspI) general secretion pathway protein I [T2SS (VF0333)] [Shigella dysenteriae Sd197] |
| Chromosome_1 | *gspJ* | 100 | 94.39 | (gspJ) general secretion pathway protein J [T2SS (VF0333)] [Shigella dysenteriae Sd197] |
| Chromosome_1 | *gspK* | 100 | 94.48 | (gspK) general secretion pathway protein K [T2SS (VF0333)] [Shigella dysenteriae Sd197] |
| Chromosome_1 | *gspL* | 100 | 93.26 | (gspL) general secretion pathway protein L [T2SS (VF0333)] [Shigella dysenteriae Sd197] |
| Chromosome_1 | *gspM* | 96.93 | 93.42 | (gspM) general secretion pathway protein M [T2SS (VF0333)] [Shigella dysenteriae Sd197] |
| Chromosome_1 | *kpsM* | 100 | 91.12 | (kpsM) KpsM [K1 capsule (VF0239)] [Escherichia coli O18:K1:H7 str. RS218] |
| Chromosome_1 | *kpsD* | 100 | 99.52 | (kpsD) KpsD [K1 capsule (VF0239)] [Escherichia coli O18:K1:H7 str. RS218] |
| Chromosome_1 | *sat* | 100 | 99.51 | (sat) Aecreted auto transpoter toxin [Sat (VF0231)] [Escherichia coli CFT073] |
| Chromosome_1 | *iutA* | 99.96 | 88.36 | (iutA) ferric aerobactin receptor precusor IutA [Aerobactin (VF0229)] [Escherichia coli CFT073] |
| Chromosome_1 | *iucD* | 98.8 | 96.3 | (iucD) L-lysine 6-monooxygenase IucD [Aerobactin (VF0229)] [Escherichia coli CFT073] |
| Chromosome_1 | *iucC* | 100 | 98.11 | (iucC) aerobactin siderophore biosynthesis protein IucC [Aerobactin (VF0229)] [Escherichia coli CFT073] |
| Chromosome_1 | *iucB* | 100 | 99.79 | (iucB) aerobactin synthesis protein IucB [Aerobactin (VF0123)] [Shigella flexneri 2a str. 301] |
| Chromosome_1 | *iucA* | 100 | 99.83 | (iucA) aerobactin synthesis protein IucA [Aerobactin (VF0123)] [Shigella flexneri 2a str. 301] |
| Chromosome_1 | *papI* | 100 | 94.87 | (papI) regulatory protein PapI [P fimbriae (VF0220)] [Escherichia coli CFT073] |
| Chromosome_1 | *papB* | 99.68 | 98.41 | (papB) regulatory protein PapB [P fimbriae (VF0220)] [Escherichia coli CFT073] |
| Chromosome_1 | *papX* | 100 | 97.64 | (papX) PapX protein regulates flagellum synthesis to repress motility [P fimbriae (CVF425)] [Escherichia coli CFT073] |
| Chromosome_1 | *fyuA* | 100 | 99.9 | (fyuA) pesticin/yersiniabactin receptor protein [Yersiniabactin (VF0136)] [Yersinia pestis CO92] |
| Chromosome_1 | *ybtE* | 100 | 99.75 | (ybtE) yersiniabactin siderophore biosynthetic protein [Yersiniabactin (VF0136)] [Yersinia pestis CO92] |
| Chromosome_1 | *ybtT* | 100 | 99.5 | (ybtT) yersiniabactin biosynthetic protein YbtT [Yersiniabactin (VF0136)] [Yersinia pestis CO92] |
| Chromosome_1 | *ybtU* | 100 | 99.64 | (ybtU) yersiniabactin biosynthetic protein YbtU [Yersiniabactin (VF0136)] [Yersinia pestis CO92] |
| Chromosome_1 | *irp1* | 100 | 99.68 | (irp1) yersiniabactin biosynthetic protein Irp1 [Yersiniabactin (VF0136)] [Yersinia pestis CO92] |
| Chromosome_1 | *irp2* | 100 | 99.51 | (irp2) yersiniabactin biosynthetic protein Irp2 [Yersiniabactin (VF0136)] [Yersinia pestis CO92] |
| Chromosome_1 | *ybtA* | 100 | 99.48 | (ybtA) transcriptional regulator YbtA [Yersiniabactin (VF0136)] [Yersinia pestis CO92] |
| Chromosome_1 | *ybtP* | 100 | 99.56 | (ybtP) lipoprotein inner membrane ABC-transporter [Yersiniabactin (VF0136)] [Yersinia pestis CO92] |
| Chromosome_1 | *ybtQ* | 100 | 99.56 | (ybtQ) inner membrane ABC-transporter YbtQ [Yersiniabactin (VF0136)] [Yersinia pestis CO92] |
| Chromosome_1 | *ybtX* | 100 | 99.45 | (ybtX) putative signal transducer [Yersiniabactin (VF0136)] [Yersinia pestis CO92] |
| Chromosome_1 | *ybtS* | 100 | 99.85 | (ybtS) salicylate synthase Irp9 [Yersiniabactin (VF0136)] [Yersinia pestis CO92] |
| Chromosome_1 | *csgB* | 99.78 | 83.81 | (csgB) minor curlin subunit precursor curli nucleator protein CsgB [Agf (VF0103)] [Salmonella enterica subsp. enterica serovar Typhimurium str. LT2] |
| Chromosome_1 | *csgD* | 100 | 81.57 | (csgD) DNA-binding transcriptional regulator CsgD [curli fibers/thin aggregative fimbriae (AGF) (AI094)] [Salmonella enterica subsp. enterica serovar Typhimurium str. LT2] |
| Chromosome_1 | *csgF* | 99.04 | 80.76 | (csgF) curli production assembly/transport protein CsgF [Agf (VF0103)] [Salmonella enterica subsp. enterica serovar Typhimurium str. LT2] |
| Chromosome_1 | *csgG* | 100 | 83.21 | (csgG) curli production assembly/transport protein CsgG [Agf (VF0103)] [Salmonella enterica subsp. enterica serovar Typhimurium str. LT2] |
| Chromosome_1 | ***ompA*** | **99.33** | **95.79** | **(ompA) outer membrane protein A [OmpA (VF0236)] [Escherichia coli O18:K1:H7 str. RS218]** |
| Chromosome_1 | *entA* | 100 | 98.13 | (entA) 23-dihydro-23-dihydroxybenzoate dehydrogenase [Enterobactin (VF0228)] [Escherichia coli CFT073] |
| Chromosome_1 | *entB* | 100 | 99.53 | (entB) isochorismatase [Enterobactin (VF0228)] [Escherichia coli CFT073] |
| Chromosome_1 | *entE* | 100 | 99.13 | (entE) 23-dihydroxybenzoate-AMP ligase component of enterobactin synthase multienzyme complex [Enterobactin (VF0228)] [Escherichia coli CFT073] |
| Chromosome_1 | *entC* | 100 | 99.33 | (entC) isochorismate synthase 1 [Enterobactin (VF0228)] [Escherichia coli CFT073] |
| Chromosome_1 | *fepB* | 100 | 99.69 | (fepB) ferrienterobactin ABC transporter periplasmic binding protein [Enterobactin (VF0228)] [Escherichia coli CFT073] |
| Chromosome_1 | *entS* | 100 | 98.24 | (entS) enterobactin exporter iron-regulated [enterobactin (IA019)] [Escherichia coli CFT073] |
| Chromosome_1 | *fepD* | 100 | 97.84 | (fepD) ferrienterobactin ABC transporter permease [Enterobactin (VF0228)] [Escherichia coli CFT073] |
| Chromosome_1 | *fepG* | 100 | 97.68 | (fepG) iron-enterobactin ABC transporter permease [Enterobactin (VF0228)] [Escherichia coli CFT073] |
| Chromosome_1 | *fepC* | 100 | 99.14 | (fepC) ferrienterobactin ABC transporter ATPase [Enterobactin (VF0228)] [Escherichia coli CFT073] |
| Chromosome_1 | *entF* | 99.28 | 97.77 | (entF) enterobactin synthase multienzyme complex component ATP-dependent [Enterobactin (VF0228)] [Escherichia coli CFT073] |
| Chromosome_1 | *fes* | 100 | 99.17 | (fes) enterobactin/ferric enterobactin esterase [enterobactin (IA019)] [Escherichia coli CFT073] |
| Chromosome_1 | *fepA* | 100 | 98.8 | (fepA) ferrienterobactin outer membrane transporter [Enterobactin (VF0228)] [Escherichia coli CFT073] |
| Chromosome_1 | *entD* | 85.86 | 97.73 | (entD) phosphopantetheinyl transferase component of enterobactin synthase multienzyme complex [Enterobactin (VF0228)] [Escherichia coli CFT073] |
| Chromosome_1 | *fdeC* | 99.93 | 97.44 | (fdeC) adhesin FdeC [FdeC (VF0506)] [Escherichia coli O45:K1:H7 str. S88] |
| Chromosome_1 | *ykgK/ecpR* | 100 | 99.49 | (ykgK/ecpR) regulator protein EcpR [ECP (VF0404)] [Escherichia coli O157:H7 str. EDL933] |
| Chromosome_1 | *yagZ/ecpA* | 100 | 98.3 | (yagZ/ecpA) E. coli common pilus structural subunit EcpA [ECP (VF0404)] [Escherichia coli O157:H7 str. EDL933] |
| Chromosome_1 | *yagY/ecpB* | 100 | 98.06 | (yagY/ecpB) E. coli common pilus chaperone EcpB [ECP (VF0404)] [Escherichia coli O157:H7 str. EDL933] |
| Chromosome_1 | *yagX/ecpC* | 100 | 97.58 | (yagX/ecpC) E. coli common pilus usher EcpC [ECP (VF0404)] [Escherichia coli O157:H7 str. EDL933] |
| Chromosome_1 | *yagW/ecpD* | 100 | 99.09 | (yagW/ecpD) polymerized tip adhesin of ECP fibers [ECP (VF0404)] [Escherichia coli O157:H7 str. EDL933] |
| Chromosome_1 | *yagV/ecpE* | 99.74 | 97.88 | (yagV/ecpE) E. coli common pilus chaperone EcpE [ECP (VF0404)] [Escherichia coli O157:H7 str. EDL933] |
| Chromosome_1 | *fimH* | 100 | 97.59 | (fimH) FimH protein precursor [Type 1 fimbriae (VF0221)] [Escherichia coli CFT073] |
| Chromosome_1 | *fimG* | 100 | 98.21 | (fimG) FimG protein precursor [Type 1 fimbriae (VF0221)] [Escherichia coli CFT073] |
| Chromosome_1 | *fimF* | 100 | 99.06 | (fimF) FimF protein precursor [Type 1 fimbriae (VF0221)] [Escherichia coli CFT073] |
| Chromosome_1 | *fimD* | 100 | 98.9 | (fimD) Outer membrane usher protein fimD precursor [Type 1 fimbriae (VF0221)] [Escherichia coli CFT073] |
| Chromosome_1 | *fimC* | 100 | 98.62 | (fimC) Chaperone protein fimC precursor [Type 1 fimbriae (VF0221)] [Escherichia coli CFT073] |
| Chromosome_1 | *fimI* | 100 | 98.52 | (fimI) Fimbrin-like protein fimI precursor [Type 1 fimbriae (VF0221)] [Escherichia coli CFT073] |
| Chromosome_1 | *fimA* | 99.83 | 90.76 | (fimA) Type-1 fimbrial protein A chain precursor [Type 1 fimbriae (VF0221)] [Escherichia coli CFT073] |
| Chromosome_1 | *fimE* | 100 | 98.83 | (fimE) Type 1 fimbriae Regulatory protein fimE [Type 1 fimbriae (VF0221)] [Escherichia coli CFT073] |
| Chromosome_1 | *fimB* | 100 | 97.84 | (fimB) Type 1 fimbriae Regulatory protein fimB [Type 1 fimbriae (VF0221)] [Escherichia coli CFT073] |
| Chromosome_1 | *aslA* | 100 | 98.18 | (aslA) putative arylsulfatase [AslA (VF0238)] [Escherichia coli O18:K1:H7 str. RS218] |
| Plasmid_A | *senB* | 99.91 | 99.74 | (senB) enterotoxin [ShET2 (VF0258)] [Shigella flexneri 2a str. 301] |
